# Neuromodulation and a Reconceptualization of Autism Spectrum Disorders: Using the Locus Coeruleus Functioning as an Exemplar

**DOI:** 10.3389/fneur.2018.01120

**Published:** 2018-12-19

**Authors:** Eric B. London

**Affiliations:** Institute for Basic Research in Developmental Disabilities, Staten Island, NY, United States

**Keywords:** locus coeruleus, norepinephrine, noradrenergic, neuromodulation, autism, autism treatment, midbrain, prefrontal cortex

## Abstract

The Autism Spectrum Disorders (ASD) are a heterogeneous group of developmental disorders. Although, ASD can be reliably diagnosed, the etiology, pathophysiology, and treatment targets remain poorly characterized. While there are many atypical findings in anatomy, genetics, connectivity, and other biologic parameters, there remains no discreet hypothesis to explain the core signs as well as the very frequent comorbidities. Due to this, designing targets for treatments can only be done by assuming each symptom is a result of a discreet abnormality which is likely not the case. Neuronal circuity remains a major focus of research but rarely taking into account the functioning of the brain is highly dependent on various systems, including the neuromodulatory substances originating in the midbrain. A hypothesis is presented which explores the possibility of explaining many of the symptoms found in ASD in terms of inefficient neuromodulation using the functioning of the locus coeruleus and norepinephrine (LC/NE) as exemplars. The basic science of LC/NE is reviewed. Several functions found to be impaired in ASD including learning, attention, sensory processing, emotional regulation, autonomic functioning, adaptive and repetitive behaviors, sleep, language acquisition, initiation, and prompt dependency are examined in terms of the functioning of the LC/NE system. Suggestions about possible treatment directions are explored.

## Introduction

The characterization and nomenclature for disorders of the central nervous system have proved to be highly challenging and even to this day, not optimally successful (1). It was not until the 1980's that a standardized nomenclature was accepted for psychiatric disorders in the form of the DSM 3 (2, 3). This was done at least partially out of the necessity to have a nomenclature which would serve the growing amount of biomedical information and treatments which were being identified. The brain, being unique in terms of its complexity and the specialization of each cell, does not permit easy characterization of pathology or even of “neurotypicality.” The DSM 3 solved the nomenclature dilemma of psychiatry by using observable behavioral symptoms as the criteria for categorization. While this solves the reliability of diagnosis issue, it sacrifices the ability to use diagnosis as a correlate of etiology. This was known and written about by the framers of the DSM 3, but frequently forgotten or disregarded over the almost half century since (4, 5). Along with the lack of etiologic understanding of psychiatric disorders, the contributions of pathophysiology, psychosocial, and developmental issues of the symptoms observed remain incomplete and often inadequate to contribute to a successful rational treatment approach. The DSM 4 and 5 have revised the diagnostic criteria of ASD with changes including the elimination of sub diagnoses such as Asperger's disorder, and Pervasive Diagnostic Disorder not Otherwise Sepecified; and reducing the criteria from three to two areas (social communication and restricted and repetitive behaviors eliminating language disorders as a separate criteria), It has more precisely defined the components of these two areas and in doing so has reduced the number of cases qualifying for the diagnosis (6), Despite this, later iterations of the DSM conform to the original concept of using observational symptoms and continue with the same liabilities related to defining etiologic, pathophysiologic, psychosocial, or developmental issues needed to understand the pathology (7).

Many of the treatments that we currently have in psychiatry were discovered serendipitously and ironically, when we attempt to use rationally developed targets, the failure rate has been unacceptably high (8–10). The recognition of these issues, led to the adaptation of the Research Domain Criteria (RDoC) program discouraging diagnosis in favor of studying observable symptoms along with brain systems or circuits (11, 12), as well as the NIMH's Experimental Medicine Initiative (9) which focuses on first finding biomarkers rather than behavioral effect. To appropriately conceptualize the disorders, a synthesis of the psychosocial and developmental contributions along with an understanding of pathophysiology can bridge the enormous gap between behaviorally based symptoms and etiology.

In the case of the Autism Spectrum Disorders (ASD), one would be hard pressed to concisely describe the etiology, developmental progression, brain pathology, or the pathophysiology (7, 13). Many brain structures and systems in ASD have been studied and found to be aberrant in structure or function compared to typically developing individuals. This long list includes the cerebellum (14, 15), the amygdala (16), the insula (17), the basal ganglia (18, 19), and various other cerebral structures (20, 21) each with compelling evidence to associate these findings with the symptoms seen in ASD. Others have described cellular or more distributed systems rather than anatomical structures, such as mirror neurons (22) or default-mode network (23, 24). The implicit assumption behind this line of research is that specific behavioral impairments can be associated with dysfunctions in particular brain areas or modules (25).

An alternative concept has been advanced by Muller (26). The multiplicity of observed pathology as well as the failure to find a unifying hypothesis has led to the suggestion that ASD should be conceptualized as a distributed disorder and that many, if not all, functional brain networks are affected. These networks can be affected during early development due to genetic, epigenetic, or by trophic activity leading to developmental anomalies later in life. A large component of the recent research in ASD has focused on the concept of connectivity. However, this term is used to describe a multi-faceted concept which varies according to the method used to study it (27), with both under, and over connectivity reported based on connection length, topological issues, and developmental issues. There has been consistency in the findings of long range underconnectivity with a more complicated picture around the concept of short range overconnectivity, nonetheless, it is noted that the variability of the methods used in research makes it difficult to come to any firm conclusion (27). Falahpour et al. (28) used a “dynamic” scanning analysis which is sensitive to physiologic change during the scanning period, as opposed to a static method which was uniformly used in the previous literature. They found that reduced “connectivity” was correlated, both in ASD and typically developing groups, to the amount of variability of signal across time within a given subject. Further, the peak connectivity in ASD was not significantly reduced in ASD. Falahpour et al. (28) could reproduce the previous connectivity findings using static methods. This led to the intriguing hypothesis that the connections are not actually “broken” in ASD but rather, subject to greater intra-individual variability across time. This is consistent with the understanding that a connectivity diagram is only the beginning, but not an answer to, brain functioning (29). Similarly, in an analysis of attention functioning, Burack et al. (30) makes the case that in ASD the results found in studies looking at local vs. global attention, are different than in neuro-typical controls, however the ability to see things locally or globally was in in itself not impaired. In ASD different circuitry might be used or used preferentially, and so ASD functioning would likely be dependent on the exact task, the timing of the task, the state of the individual at the time of the task, the environment in which the task was presented (distractors or not) and many other variables. All of these factors might affect and be affected by neuromodulators. The “hard wiring” without an understanding of the neuromodulatory effects on that circuit, describes only a fraction of the potential or functional capacity of that circuit. Stated another way, many of the symptoms seen in ASD might be state conditions rather than invariable traits, and treatments which focus on normalizing the state of the individual could have the most potential for success.

The goal of this paper is to suggest that much of what we know to be the symptomatology of ASD, can be conceptualized as due to inadequate regulation of brain circuitry with a specific focus on one of the main neuromodulators, the locus coeruleus/norepinephrine (LC/NE) system. Theories concerning the LC/NE and its functioning have been developed over the past few decades. Several authors have written treatises on this topic (31–36). Despite much indirect evidence of the LC/NE involvement in ASD (to be presented in this paper), there is little direct evidence. The size, shape, and location of the LC make it challenging to target for ablation inhibitions studies. Another difficulty is with its extensive projection pattern such that that manipulations of the LC affect NE signaling in many parts of the brain. These problems are present in animal model studies and the difficulty in studying LC/NE functioning in the human brain is that much more difficult. The LC itself may or may not have demonstrable “lesions.” Attempts to directly test the association of components this system have been very limited. The norepinephrine transporter gene (SLC6A2) was found not to be associated with the diagnosis or the phenotype of ASD although the authors noted that no other genetic markers of noradrenergic functioning were tested. In a Letter to the Editor, Martcheck et al. report on direct postmortem study of 5 cases of ASD compared to 5 control cases and found no difference in the total cell count, the volume of the LC, and the numerical density (37, 38). This scant evidence does little to contradict what appears to be an overwhelming amount (albeit indirect) of associations with the functioning of this system and ASD. These neuromodulatory systems, derived from brainstem and midbrain nuclei, connect with all parts of the brain. Neurochemically there are several neuromodulators which are likely to play a significant role in ASD including serotonin, dopamine, acetylcholine, norepinephrine, and others (36), and the case for the involvement in the LC/NE system in no way precludes the important role of other neuromodulatory systems. As an example hyperserotonemia is present in 25% of children with ASD and there are multiple hypotheses of how serotonin may interact with other substances to produce the changes found in ASD, at least in a subset of cases (39). Brains of ASD individuals have abnormalities in both serotonin transporter and dopamine transporter binding (40) and based on various findings in ASD have led to hypotheses which suggest dopamine functioning is central to the understanding of ASD pathology (41, 42). These neuromodulators appear to function synergistically, innervating the other neuromodulatory nuclei (as well as cortical areas) and so a complete understanding ought to include all of the neuromodulatory systems (34, 43, 44). Due to the enormous complexity of all these modulator systems on the whole brain, for practical consideration I will focus only on norepinephrine and its center, the locus coeruleus, which appears to have a prominent role in many of the symptoms associated with ASD.

In an effort to reframe the conceptualization of ASD I propose the following hypotheses:

1. Much of the pathology observed in ASD is due to inadequate regulation of circuits rather than intrinsic flaws in the circuitry themselves and the activating and deactivating of circuitry is a central function of the LC/NE system. In ASD the failure to adequately regulate the functioning of the circuitry inhibits the brain's ability to make functional behavioral responses to the ever-changing environment. This will be true regardless of the etiology of autism (idiopathic or syndromic).
2. The LC/NE system plays a central role and explains much of the dysregulation which create the symptoms present in ASD, although other neuromodulators play and important role as well.
3. To explain these phenomena, we need to describe a broad range of neurons and circuits. The pathology in ASD, being a distributed one rather than a localized one with an enormous number of brain functions being atypical in ASD, mirrors the distributed nature of the LC/NE system.
4. Flaws in the functioning of the LC/NE system are likely not due to primary pathology in the LC neurons themselves, but rather might be secondary to afferent input to the LC or a deficit in the circuitry which LC efferents are meant to stimulate.
5. These hypotheses needs to be understood as dysregulation rather than hyper or hypo functioning. Many symptoms of ASD can be characterized as functional deficits (rather than hard lesions) and are dependent on the state of the subject at the time of testing. ASD could be conceptualized as a disorder largely due to a failure of homeostatic mechanisms.
6. Interventional strategies aimed at normalizing the neuromodulatory functions of the LC/NE system will be a plausible and promising target for effective treatment strategies and need to be a high priority for treatment research.

To explore the above hypotheses, we will first review the basic neuroscience of neuromodulation with an emphasis on norepinephrine. We will then review the various symptoms associated with ASD and examin of how the LC/NE system could be involved. Finally, we will discuss the treatment possibilities available concordant with a neuromodulatory hypothesis.

## Neuromodulation

Neuromodulation is an enormous topic (45). Due to its lack of localization and sheer complexity, it is difficult to precisely integrate these phenomena with human pathology, nevertheless, its importance is undeniable. The neuromodulators also function as neurotransmitters (causing direct excitatory or inhibitory effects on postsynaptic neurons), however their other function is to regulate and modulate the neuronal effect of other neurotransmitters (35, 36). The infinitely changing environment with which organisms need to cope, calls for a similar degree of flexibility in order to adapt to it and neuromodulation is the mechanism to accomplish maximal flexibility of the central nervous system (CNS). Neuromodulators can be distributed via the bloodstream, via volume transmission (extracellular fluid) as well as having widespread release sites such as synaptic varicosities. Massive axonal arborizations have huge numbers of release sites (46–48). On a neurofunctional level, neuromodulation can aid in hyperpolarizing or depolarizing neurons, changing their responsivity to input, altering the strengths of synapses, and shaping the plasticity of those synapses. All of this can influence long term potentiation and depression and in harmony with neural activity, mediate plasticity (48). Input from a small number of neuromodulatory cells can abruptly interrupt the activity of neural networks and reorganize the elements into new functional networks. A single neuron can participate in several networks and a single anatomical network can mediate multiple functions (32).

Adaptive behavior in a diverse and changing world requires a tradeoff between exploiting the known sources of reward and exploring the environment for other potentially more valuable or stable opportunities. The brain mechanisms which can perform this function trade off complexity required for a broad and flexible repertoire of behaviors with the efficiency of function that comes with a simpler design. In their Adaptive Gain Theory, Aston Jones and Cohen (35, 36), propose how the LC/NE system may be central to these functions. Classically, this system was associated with arousal which plays a role in behavior including sleep, attention, anxiety, stress, and motivation. Dampened arousal leads to drowsiness and ultimately sleep. Heightened arousal is brought on typically by an emotionally salient event and facilitates functional behaviors in response. Optimal performance is associated with an intermediate level of arousal and worsens with too little or too much arousal, described by the classic Yerkes-Dodson curve or an inverted U shaped curve (36).

## The Locus Coeruleus and Its Connections

**Figure 1:**
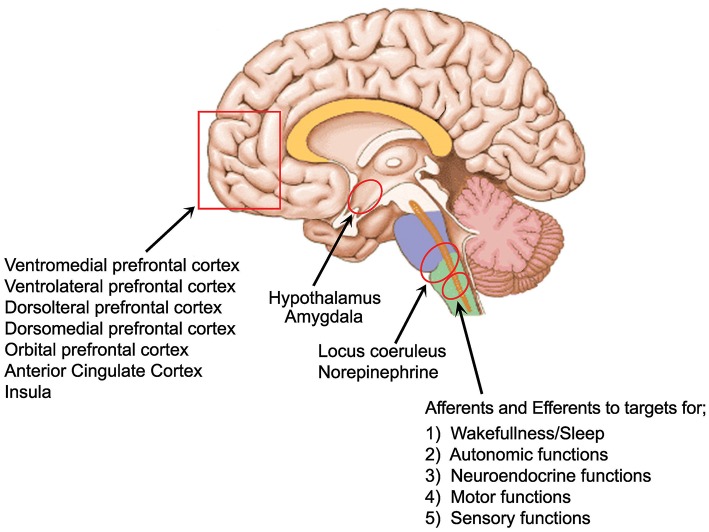
Areas of strong LC connection and of interest to ASD.

The LC, which is the largest NE nucleus in the brain, projects to the entire central nervous system (see Figure 1) modulating sensory processing, motor behavior, arousal, and cognitive processes (49). Ironically, it is one of the smallest nuclei comprising around 1,600 cells with a common embryonic origin (50). It receives afferent information from a multitude of regulatory centers. These areas include the prefrontal cortex, the bed nucleus of the stria terminalis, hypothalamic nuclei, raphe nuclei, amygdala, solitary nucleus, nucleus paragigantocellularis, and the nucleus prepositus hypoglossus, indicating that it integrates information from the autonomic nervous system, neuroendocrine nuclei, stress, and limbic circuitry as well as higher order cognitive centers (49). There is also abundant input from motor related nuclei in the midbrain, pons, medulla and cerebellum (51). Various structures connect to various parts of the LC. As examples, with retrograde tracing it was found that the hippocampus and septal nucleus communicate with the dorsal portion of the LC while motor related structures such as the caudate-putamen and the cerebellum labeled both the ventral and dorsal portions. Hypothalamic injections labeled cells in the anterior pole and the amygdala and cortical structures produced labeling of neurons scattered throughout all three axes of the compact core of the nucleus (49). Deep cerebellar nuclei and cerebellar Purkinje cells contribute a significant fraction of direct synaptic input to LC/NE neurons (51). They further found that the Purkinje cells contribute input to LC/NE population that do not project axons extensively to any of the output sites examined, suggesting these inputs are predominantly regulatory to the LC. A deficiency of Purkinje cells has been found in 75% of the ASD cases studied in post mortem research, the most consistently reported biologic finding in ASD using that method (52, 53) and these cells appear to have a special role in regulating the LC.

The output connections or the LC are widespread and divergent. The LC/NE neurons project to the olfactory bulb, auditory cortex, hippocampus or medulla as well as 7 other sites studied by Schwartz et al. (51). LC/NE afferents are present throughout virtually the entire forebrain. All layers of the cortex are innervated but with varying anatomic characteristics. Based on the density of varicosities and mRNA expression, it appears that some areas of the cortex are more highly innervated (49). This innervation has been found to be higher in the frontal cortex, compared to the motor somatosensory and piriform cortices, with the highest found in the Anterior Cingulate Cortex (ACC) (54). Although the prefrontal regions receive the most NE stimulation, the insular cortex uniquely is innervated by both the LC and non-LC derived NE containing fibers, suggesting that the insula may play a role in unique sensory or environmental circumstances (55). The critical role that NE plays in prefrontal cortical (PFC) functioning implicates it in a variety of neuropsychiatric and neurodegenerative diseases which are characterized by deficits in the PFC (49).

Taken as a whole, the functioning of the LC/NE system needs to be considered as a dynamic equilibrium with both feedback and output often going to the same anatomic areas. Conceptualizing the LC as anything other than a component of a larger system misses the central point of its primary function being neuromodulation of the entire CNS.

## Physiology of the LC

The LC fires both tonically and phasically and to understand its functioning it is important to consider both. Phasic discharges are characterized by brief 10–20 Hz bursts of two to three action potentials which is often followed by a sustained suppression of spontaneous activity lasting 200–500 ms. These phasic bursts are elicited by novel or salient stimuli as well as top down decision signals mostly from prefrontal cortical regions. These bursts are associated with task related decision processes and are related to ongoing evaluations of task utility, or the cost and benefits of a particular behavior. As the utility of that behavior wanes (for example reaching satiation), tonic activity of the LC/NE takes over, prompting behavior designed to explore the environment for other sources of reward (35, 36).

This function of the tradeoff between a focus on a functional task and non-focused scanning of the environment, could be thought of as a fundamental issue in autism. In ASD, the repetitive behaviors which are not goal oriented and are not rewarding (at least in a typical framework) are performed when neurotypical individuals would have reached satiation and moved into a scanning mode to seek out more rewarding pursuits through detachment from the original focus. At other times, individuals with ASD appear not to be focused, and even with the presentation of what appears to be a rewarding stimulus (for example a toy to a child), there is no move to focus attention on that stimulus. In both cases it could be speculated that there is a lack of phasic discharge to alter the circuitry and allow transition.

Phasic LC output likely increases sensory signal transmission through thalamic and cortical circuitry. During phasic firing, redundancies across the circuitry which are being facilitated are believed to enable a reduction in noise thus enhancing the effect of small fluctuations in stimulus intensity which promotes the sensitivity to sensory input. This in turn facilitates the engagement and focus on a given task. For example, a mouse which discovers food but needs to learn a complicated task to get it must figure out the strategies needed to obtain the food which requires intense focus, and attention to small details which otherwise would not be otherwise salient. In addition, a disregard of the larger environment is needed to minimize distraction. In ASD it has been long noted that there is an “incredible” ability to focus on detail (30) and when extreme, it may facilitate the creation of a savant skill. If a phasic stimulation induced event was to be prolonged (more accurately, not overridden), a microscopic focus with incredible detail might take place on an interest which typically would not be thought of as salient. In neurotypical individuals there would be a balance of focus with scanning. This could also be framed as the cause of obsessive preoccupations with a limited number of objects which is a core sign of ASD.

Tonic rates of firing correlate with arousal levels within the sleep/waking continuum (56). Optimal tonic output facilitates transmission of sensory signaling so that low level and high level sensory input are both capable of detection, extending to marginal, and even subliminal inputs. The adaptive gain model (35) predicts that tonic LC activity increase the “gain” of global responsivity of cells in noradrenergic terminal fields to any excitatory input, facilitating scanning. Phasic LC output, on the other hand increases the “gain” of all units in a manner that produces an “all or nothing” response profile (56) and weakly active neurons are suppressed concordant with a competitive enhancement of strongly active neurons facilitating a specific focus and a reduction of distraction.

Optimal phasic LC response occurs when the tonic firing is in the moderate range (35, 36). Under conditions of stress, the tonic LC firing is increased, which suppresses phasic responses from the LC (57). This is consistent with the evolutionary role of keeping the orgasm in a scanning mode when there is danger present. This could also be consistent with the situation in ASD in which there is overabundance of time in the stress mode (which may be supported by some evidence such as overactive sympathetic activity and behavioral correlates such as hyperactivity and impulsivity), leading to a higher rate of tonic firing and a less responsive phasic mode. While this is an intuitively attractive hypothesis for ASD, it is difficult to prove in humans. One line of evidence for this is that children with ASD were found to have higher baseline pupil size (58, 59), and there is evidence that pupil size is a good measure for LC activity (60, 61). High tonic LC activity would inhibit phasic response. If this is the case, the reason for the dysfunctional balance of tonic to phasic functioning would be an important question to ask. At this point, we know that corticotropin releasing factor (CRF) stimulates the LC and its overstimulation can produce a high tonic condition while endogenous opioids have the opposite effect promoting lower tonic tone. The regulating of tonic firing rates with the above mentioned substances or other yet unexplored mechanisms will affect the phasic functioning of the LC (57).

As explained, during periods of elevated tonic LC activity, the ability to discriminate targets from distractors and the threshold for responding to stimuli are decreased (35). Temple Grandin (a high functioning individual with ASD) describes her inability to speak on the phone if she is in a noisy environment due to an inability at filtering out noise. “I am unable to talk on the phone in a noisy office or airport. Everybody else can use the phones in a noisy environment, but I can't. If I try to screen out the background noise, I also screen out the phone” (62). Educational approaches to ASD have long noted the need to keep the child's learning environment as free from distractors as possible (63). A typical classroom (or nearly any other socially integrated setting) is not free of distractors, creating a conundrum for the educator between optimizing the learning ability of the ASD student vs. the necessity of introducing the student to the real-world predicament of unexpected distractions. To date, the need to biologically address this issue as a treatment focus has not received significant attention from the research community.

## Learning

Although not in the definition of the ASD, there can be little doubt that a deficit in learning is at the heart of the disorder. Children are not born with social skills, rather they need to be learned. In developing the science of Applied Behavior Analysis for learning and treatment of ASD, Ivar Lovass and others noted that these children were largely unsuccessful at learning from the natural environment and thus the environment should promote simplified instruction and potent reinforcers (64). Most of the techniques used have been derived from the principle of “stimulus control,” that is, the presence of a specific stimuli as a result of prior reinforcement. Stimuli which are effective for typical learners, such as spoken requests and modeling, are not as effective in learners with ASD (65). The question of why individuals with ASD have difficulty learning, especially in the typical manner, remains a question a half century after it was noted. The reasons for learning difficulties in the various psychiatric disorders is not likely to be unitary; therefore the neurobiology of learning difficulties in the Learning Disabled, the Intellectually Disabled, and the ASD may overlap in some respects and there may be variation within each disorder. Well-described simple models, such as single neuron synaptic formation with long term potentiation is no doubt central to learning: however in more complex organisms, this is only the tip of the iceberg. While a deficit in synaptic functioning cannot be overlooked, it is clearly necessary to look at more complex circuitry and how it functions. Deficits in learning can be caused by deficits in several functions including attention, memory, sensory processing, emotional dysregulation, and others. These functions will be reviewed with a focus on a potential LC/NE role in ASD.

## Attention

Deficits of attention are seen in many psychiatric diagnoses including ASD. The LC/NE system plays a prominent role in attention. Attention is defined as information processes that mediate perceptual selection (66). It is composed of both (1) an endogenous top down, goal directed process that is dependent on desires, expectations and or knowledge and (2) an exogenous bottom up process integrating sensory information through lower centers, which may be at odds with goal directed behaviors. Attention consists of three independent attention networks: alerting, orienting and executive control. Alerting refers to the state of awareness of the environment, orienting directs attentions to the specific sensory stimuli or location, and executive control refers to the resolution of conflicting attentional information (67). All three are dependent on LC/NE functioning. NE action in the thalamus and cortex strongly influences arousal and behavioral states (68) as well as gating and tuning influences on sensory processing (56, 69). In the frontal cortex, NE has been shown to be critical for working memory and focusing of attention. NE plays an important role in attentional shifting and behavioral flexibility (32, 36, 70).

Impairments of attention have been described as associated or comorbid with ASD (71). Taken further, these attentional deficits in ASD have been shown to be associated with emotional regulation, and inflexibility in behavior (72) and therefore are central to the disorder. Abnormal disengagement of attention has been well-studied and is hypothesized to be a primary disturbance in ASD (72).

From a developmental prospective, early attentional disorders can have far reaching effects on cognitive development (73). Visual orienting is the earliest attentional deficit seen in the high-risk infant siblings of already diagnosed older siblings with ASD (74). These attentional impairments may cause a child to lock onto certain aspects of the visual environment and lose the ability to use environmental cues (analogous to the inability to move from phasic to tonic LC functioning in animals). This in turn could hamper his or her ability to predict and prepare for a shift in visual attention (anticipation). The child may stay focused on irrelevant input. Atypical visual processing can impede top down exploration of the visual environment and may result in delayed orienting leading to impairment of learning and development (75).

The striking observation of attention to detail in ASD led to the “weak central coherence” model (76) which noted enhanced performance on visual tasks requiring attention to detail such as embedded figures and block design. This led to the theory that global attention was impaired however this theory has been challenged because, when tested, persons with ASD did not show deficits in global processing. Another theory which emerged from these observations known as “enhanced perceptual functioning” (77) suggests that in ASD it was a matter of the style or preference rather than a difference in abilities. When global and local processing were presented in competition with each other, global processing was impaired but not when administered independently (78). Neurotypical individuals appear to balance the local vs. global processing. Network reset theory, in which the LC/NE system causes a rapid resetting of the circuits (32), offers a mechanism for this. When there is both local and global information, the intact LC/NE system facilitates rapidly switching to assess both. A poorly regulated LC/NE system may not offer this opportunity, and so leads to a functional deficit but with the potential to attend to either one or the other.

Looking at superior attentional function in ASD, using pupillometry as a proxy for LC/NE functioning, Blaser et al. (59) found greater tonic pupil dilation and a greater phasic response during a visual search task, with the larger phasic pupil response associated with better search performance. This would appear to be inconsistent with the animal research in that high tonic LC functioning inhibits the triggering of phasic response; however in ASD aberrant regulation can lead to different states. They posit a hyperphasic state, increasing performance on tasks that benefit from focused attention as well as poorer functioning on tasks which require shifts of focus. They also suggest that along with the intense attentional focus noted in ASD, there is also resistance to disengagement to certain classes of stimuli. This may be central to the restricted interests and behaviors noted in ASD.

## Sensory Processing

Altered sensory perception was described in the first scientific reports of ASD (79), and up to 95% of parents of children with ASD report some atypical sensory behavior in their child (80, 81). It wasn't until the DSM 5 that “hyper or hypo reactivity to sensory input or unusual interests in the sensory aspects of the environment” was recognized as core diagnostic symptoms of ASD. As is true with many of the measured symptoms of ASD, sensory impairment is not independent of the other findings in ASD (81). Early sensory sensitivities predict attention and language development (82), development of social play (83), increased withdrawal and negative temperament (84) and higher levels of social impairment (85). The atypicality in ASD extends to all five senses, vision, auditory, touch, smell, and taste [see review by Thye et al. (81)]. Although early sensory sensitivities and later behaviors has been well-documented, research documenting a causal connection is lacking.

Sensory input can be broken down into three components; sensation, perception and attention (81). Perception involves the interpretation of the sensory input which will involve more coordination of circuity. Attending at the correct time, needed for attention, involves even greater levels of coordination. The variability of sensory perception is consistent with the known functioning of the LC/NE. LC stimulation modulates single neurons and neuronal circuitry which affect sensory perception (86) with differential responsivity mediated by levels of LC stimulation (56, 69).

A study of response reliability of the visual, auditory and somatosensory systems (25), found no differences in mean response amplitudes between ASD and controls; however the trial to trial variability was significantly larger in ASD for all three sensory modalities tested. The variability was only in those brain areas which were stimulated by a task and so the variability was not general but rather in response to the stimuli. This is concordant with a neuromodulation hypothesis in that the phasic stimulation elicits a response, but if the LC/NE system is not reliable, the circuitry needed for perception and attention will appear to be unavailable at times compared to neurotypicality in which the circuits will reliably respond to the sensory input. These findings were consistent with other reports (28, 87, 88). The similar findings for all sensory modalities suggests a distributed abnormality rather than malfunctioning of a specific system. The observation of signal variability has often been relegated to being termed noise; however theorists are exploring its meaning in that an under-variable signal might lead to stereotyped or rigid responses while an over-variable signal might lead to a lack of reliable recruitment of valuable learned strategies to adjust to the environment (89). In ASD with both overly rigid responses and a relative inability to access learned strategies (also called priors) (90), a faulty neuromodulation hypothesis is an attractive explanation.

## The LC and Emotions in Autism

The behavioral state of an organism includes mood, motivation, stress, vigilance, arousal, and attention and although these states are often studied and thought of separately, they are inextricably connected. The peripheral nervous system brings information from the environment resulting in release of hormones and activity to the neuromodulatory neurons of the midbrain and brainstem including the LC (34). Emotions are mental and bodily responses that are deployed automatically when an organism recognizes that a situation warrants such a reaction (91). Emotional regulation, recognition and communication have been described as major deficits in ASD (92–94). Emotional dysregulation leads to maladaptive responses which takes the form of irritability, poor anger control, temper tantrums, self-injurious behavior, aggression, and mood dysregulation (95). Emotional dysregulation is described in infants who later receive an ASD diagnosis and this is often seen before other symptoms (93). These early signs of predominantly negative affect persist into childhood, adolescence and adulthood with evidence of more anxiety, emotional lability, and anger than seen in neurotypical or other developmentally delayed people (96). Although not defined as a core feature of ASD, it has been found that the symptom severity of the core symptoms is related to the severity of emotional dysregulation. Restricted and repetitive behaviors have been shown to be the best predictor of emotion dysregulation (95). Cognitive functions, such as disengaging and shifting attention, while perhaps primarily impaired, may possibly be a manifestation of emotional dysregulation which then blocks task performance (93).

It is very likely that the LC/NE system is involved with the aberrant regulations of emotions in ASD and this effect is both physiologic and developmental. In a mouse model, it was found that by inhibiting NE signaling during an epoch of development (postnatal days p10–p21) it resulted in enduring changes in adult emotional behavior and changes in stress-related LC neuron activity (97). This inhibition did not cause these enduring changes during an earlier epoch (p2–p9) when the LC has limited physiologic response to the environment or during a later epoch p56–67 after the maturation of the LC and likely the circuits it forms. Also, the hippocampus and the PFC showed a robust reduction in alpha 2 A receptors when perturbed during the p10–21 period, while other structures such as the amygdala did not evidence this reduction and only the PFC exhibited a long-lasting decrease in baseline NE.

There is a great deal of variability in the ability of individuals with ASD to recognize emotions from still images. There is improved performance with prototypical facial expressions and simple emotions rather than complex emotions and the performance varies with the emotion studied (94, 98, 99) but the overall ability of individuals with ASD to recognize emotions is impaired. A study using pupillometry revealed reduced unconscious emotional reactivity in ASD but with no group differences on consciously presented emotions (100). The neurotypical group had the same responses when seeing the pictures for 2 s or for 30 ms, while the ASD group showed significantly reduced activity when shown the pictures for 30 ms (a subliminal task). The authors make the interesting case that much of the social deficit seen in ASD is because of a failure to perceive what neurotypical individuals unconsciously perceive. That is, the subtle social cues and much of our social emotional lives depend on feeling states that can lie below the threshold of consciousness. The ability to perceive subliminal emotional messages is present in neurotypical individuals as early at least as 7 months of age (101) and so this lack of ability to perceive subliminal emotional input can have a significant impact on development. In cortically blind individuals, using the subliminal task, emotionally inducing stimuli evoked a robust emotional reaction (facial, pupillary, and neural) despite a lack of conscious registration of the stimuli. Similarly, individuals with right sided parietal lobe damage who had “inattentional blindness” also did well-responding to faces with the 30 ms showing. In neurotypical individuals a “making paradigm” (showing the emotional stimuli for 30 ms and then an ambiguous or neutral face) led to no conscious awareness of the emotion, although neural and autonomic recognition of the emotional content is present (100). These phenomena have been explained by the likelihood that phylogenetically early parts of the brain are used, bypassing the cortex to elicit this fast pupillary response. The “emotional brainstem” (102) with its ascending network sends sensory information from the body to the rostral structures primarily via spinothalamic tracts. The descending network activates emotional reactions (salience) and this is via the periaqueductal gray, amygdala, and hypothalamus. The modulatory network (which would include the LC/NE systems as well as cholinergic, dopaminergic, and serotonergic input) coordinates the interaction between the other two networks. These phylogenetically older systems synthesize these emotions and then provide input to the cortical regions, most prominently the frontal, insular, and cingulate areas (again, the areas with the most NE connection in the cortex). A parsimonious explanation for the findings of Nuske et al. (100) would be the lack of synthesis of the brainstem inputs, thus requiring individuals with ASD to use alternative pathways and not able to utilize the fine-tuned and very rapid emotional responses typically seen. An inadequate LC response might break down the synchronization of this very rapid system. MEG findings of emotional faces showed a complex progression of both over and under activation of the frontal, temporal, temporal-parietal, and limbic brain regions with the “social brain” (ACC and orbitofrontal cortex) demonstrating abnormal activity in ASD (103) suggesting intact hard wired circuitry but abnormal modulation.

## The Autonomic Nervous System (ANS) and Homeostasis

Stress is the consciously or unconsciously sensed threat to homeostasis (104). The LC is reliably and robustly activated by acute stressors, both visceral (such as bladder distention, heat, and cold, etc.) and environmental, with a large literature spanning 40 years (34). This activation is via autonomic afferents from the periphery mediated by input from the ascending vagus via the nucleus tractus solitarius (NTS) (105, 106). The bidirectional ANS both sends afferents to theCNS and efferents regulating end organs such as the gastro-intestinal organs, heart, lungs, and immunes system. In ASD, there is a considerable literature concerning comorbid symptoms outside of the CNS (107). There are many symptoms, somatic, cognitive, and behavioral, which can plausibly be explained by aberrant autonomic functioning (108). Visceral organs, including the cardiopulmonary system (109, 110), the immune system (111), sleep disorders (112), gastro-intestinal disturbances (113), and impaired bone density (114) are all highly dependent on autonomic input and output for proper functioning. Research on these comorbidities appear to assume all of these to be unrelated problems but this approach has yielded little in the way of findings to explain the dysfunctions. These disorders can alternatively be conceptualized as functional or secondary to poor neuromodulation. As of now there has been little if any research to explore this hypothesis.

In the realm of the CNS, there is considerably more understanding of the relationship between the ANS and the CNS both anatomically and functionally. The central autonomic network (CAN) has been described and comprised of the anterior cingulate, insula, ventromedial prefrontal cortex, central nucleus of the amygdala, hypothalamus, periaqueductal gray matter, parabrachial nucleus, NTS, nucleus ambiguous, and other medullary areas (115). These are largely the same areas with the most exuberant connectivity to the LC. A functional neuroimaging study of individuals who had been treated for seizures with vagal nerve stimulation, found long term brain changes in the thalamus, cerebellum, orbitofrontal cortex, limbic system, hypothalamus, and medulla (116). As most of these areas are regulated by the LC, the neuromodulation function of the LC/NE system appears to be intimately involved with the functioning of the ANS, explaining many of the symptoms noted. Using neuromelanin as a proxy for LC/NE activity, it was found that there was a negative correlation between the LC activity and high frequency heart rate variability, consistent with the LC's role of inhibiting parasympathetic modulation of the heart (117). This would also fit with the hypothesis of high tonic LC/NE output (117) along with low heart rate variability (HRV) present in ASD (109, 118).

There is evidence that in ASD there is an imbalance of autonomic functioning with an overactive sympathetic system and a blunted parasympathetic system (119). Measurements thought to be proxies for parasympathetic functioning show low heart rate variability or a similar measurement respiratory sinus arrhythmia (RSA). The Polyvagal Theory correlates blunted parasympathetic functioning with deficits in several biologic components of social engagement and receptive language skills (118). Both auditory processing and RSA improved together after an intervention which stimulated the facial and trigeminal nerves via exercising the middle ear muscles (120) which is part of the social engagement system. While the functioning of the ANS is more amenable to measurement, the functioning of that system is highly regulated by the LC/NE system (121). Therefore, the LC/NE system may be involved with the many known autonomic anomalies found in ASD.

## Adaptive Behavior and Restricted Repetitive Behaviors (RRB)

One of the two diagnostic symptoms associated with ASD according to the DSM 5 is restricted repetitive patterns of behavior, interests, or activities (RBB). This category comprises stereotyped or repetitive motor movements, insistence on sameness, inflexible adherence to routines, highly restricted fixated interests, and hyper or hypoactivity to sensory input. This has been grouped into three categories; repetitive motor behaviors, insistence on sameness, and circumscribed interests (122) which clearly must have multiple pathophysiologies and therefore etiologies (123). Complicating an already difficult issue, there is little consensus on terminology with respect to the repetitive behaviors seen in association with ASD. A given act such as hand flapping may be described as stereotypic, self-stimulatory, ritualistic, perseverative, gesturing, or posturing by different clinicians. Similarly, terms such as abnormal preoccupations, circumscribed interest patterns, abnormal object attachments, and idiosyncratic responses to sensory stimuli often lack specific behavioral referents (124). Repetitive behaviors occur in a wide variety of developmental disabilities (e.g., intellectual disability), psychiatric disorders (e.g., schizophrenia, obsessive compulsive disorder [OCD]), and neurological conditions (e.g., Parkinson disease, Sydenham chorea, Tourette syndrome) (124, 125). As such, that the restricted and repetitive behaviors are not specific, nor do they represent a single biologic phenomenon. The research on the neurobiology of these symptoms is scant and in order to treat these troubling symptoms a better biologic understanding of the symptoms is needed (126).

The functioning or malfunctioning of the LC/NE can offer a novel explanation for many of the symptoms observed. Adaptive behavior in a diverse and changing word requires a trade-off between exploiting known sources of reward and exploring the environment for other opportunities. The capacity to support the adaptive behaviors requires a flexibility of repertoire (the opposite of RRB) and is thought to be primarily mediated by the LC/NE system (36). As increasingly valuable states are identified, and considering the cost of the behavior, input from the orbital frontal cortex and other cortical areas guide the brains decision of how to behave or act. Once the reward is maximized after exploiting the environment, the organism needs to shift back to exploring. This attentional shifting or set shifting is dependent on the LC/NE system (49, 127–129). The Biased Attention via Norepinephrine Model (130) describes in more detail how the more salient stimuli compete for attention. The LC/NE system interacts in these processes at multiple levels. One level would be that LC is involved in the associative learning of what is salient (131). In ASD salience is clearly different than in neurotypicals and preoccupation with bizarre fascinations (a term widely used in the Asperger's Disorder literature) can be explained by impaired associative learning of what would be salient, with the word bizarre implying that others (perhaps with an intact LC/NE system) would have a very different set of salient interests. Based on the emotional processing issues described above, rather than a natural set of emotional driven memories guiding salience, it is possible that a dysregulated LC/NE (most likely a hyperfunctioning of NE output) simulates idiosyncratic salient situation and consolidates those memories into long term memory, making those memories the template for salience. The LC/NE is central for the process of consolidation and reconsolidation of memory. Indeed clinicians who treat ASD are very aware that directing away from their salient preoccupations is not an emotionally neutral task but is very intertwined with affective systems and, in many cases, elicits extreme responses such as aggression.

## Sleep

The tonic activity of the LC/NE system, strongly covaries with the stages of the sleep-waking cycle, firing most rapidly during waking, slows during drowsiness and slow wave non-REM sleep and become virtually silent during REM sleep. Low levels of LC activity facilitate sleep and disengagement from the environment (36).

Sleep has consistently been found to be disturbed in individuals with ASD significantly more than in the neurotypical population, with sleep disturbance reports ranging from 44% to 83% prevalence in this population based on parent report (132–134). Buckley et al. (135) found shorter sleep time, greater slow wave (stage 3) sleep percentage, and much smaller REM sleep percentage compared to neuro-typical subjects. Comparing ASD to children with developmental delay, there was shorter total sleep time, greater stage 1 sleep percentage, and greater slow wave sleep percentage. Studies in people with autism have identified various abnormalities in REM sleep including immature organization, decreased quantity, abnormal twitches, undifferentiated sleep and REM sleep behavior disorder, which is characterized by the absence of the muscle atonia that is normal during REM sleep. Clinically, the sleep disturbances are significant in both low and high functioning subjects (136–138). The pervasiveness of the sleep disturbances noted may be masking their significance to clinicians, who may accept this as part of ASD rather than a symptom demanding some urgency. While some symptoms are obvious to parents and clinicians, such as daytime sleepiness or behavior problems at night, there are many other symptoms which may be related to the sleep disorders which are more difficult to recognize.

Learning and memory have been shown to be dependent on sleep functions (139). Both REM and nREM sleep appear to be instrumental to the learning and memory process. A two stage memory process has been proposed originally by Buzsaki (140) in which information is gathered during wakefulness and during exploratory activity when the hippocampus activity oscillates in a theta/gamma mode. During this time, cortical information is transmitted and weakly potentiated onto CA3 neurons. During offline sleep, fast oscillations, called “ripples” with synchronous population burst 5–8 times higher than during wakefulness, result in long term potentiation of CA1 neurons which is followed by hippocampal output to the cortex. Mechanistically, noradrenergic and dopaminergic firing is thought to recruit neurons into a broad network underlying new learning and during sleep there is a replay of the memory ensembles. Animals that learned a task were shown to have the above described ripples during sleep while electrically stimulated interruption of these ripples inhibited learning (141). Memory deficit was accompanied by decoupling of coordinated hippocampal-cortical activity due to LC activation during the offline sleep periods. Gina Poe (141) proposes that when new pieces of information are learned, synaptic weakening is an important part of the reconsolidation of information. When the LC is silent, it is the only time when synapses can be weakened, allowing information to be forgotten and therefore replaced with new learning. Without this reconfiguration, new information cannot be incorporated into old schema, leaving the new learning fragmented. If this is the case, it supports the hypothesis of Pellicano et al. (90) which notes that in ASD perceptions are not integrated into “priors” or pre-existing structures. The information is then left to be dealt with, without a context, explaining much of what is seen in autism. Without the ability to “prune” and update information rigid and repetitive behaviors are a likely, providing a plausible mechanism to explain cognitive models of ASD such as poor generalization of learning and weak central coherence (76, 142).

## Initiation, Prompts, and Failure to Inhibit

Well-known deficits in ASD include difficulties in the initiation of behaviors and transitioning from one behavior to another. Both can be explained through poor functioning of the LC/NE system (36, 143). Many behavioral deficits are treated by means of “prompts.” In the behavioral literature, prompts are described as antecedent stimuli that are effective in getting responses to occur. The prompt is added to a situation in which the naturally occurring stimulus does not yet control the response (65). MacDuff et al. (65) go on to describe many types of prompts including verbal prompts, modeling, manual prompts, gestural prompts, textual prompts and others. In many cases the prompt appears to have little to do with the behavior which is elicited. An example might be a student who is unable to do a task,but, with one word or even a physical touch, might be able to initiate a chain of complex behaviors. Prompts are also known in the neurological literature, generally referred to as a “cues.” Neurotypical individuals also use prompts in their daily lives (such as smartphones buzzing to remind us of a meeting, leading to a chain of behaviors involved with attending the meeting). The distinction is that in ASD many of the daily behaviors need prompting, and the prompting becomes central to functioning. Prompting is so easy and successful in ASD treatment, that it has generated a large literature concerning how to fade or withdraw the prompts, which is often more difficult than instituting the prompt. Applied Behavior Analysis (ABA) takes a functional approach to teaching so, if the behavior or skill is not demonstrated, the operating assumption that there is a lack of mastery or acquisition. On the other hand, a cognitive skill being taught, such as color discrimination, must be rather tedious if the child “knows” the colors but cannot initiate the movement of his hand and point to the correct response. ABA therapists have adjusted to this reality with various methods such as errorless teaching using simultaneous prompting in which the prompting compensates for the deficit as part of the teaching (144). Later, when the teaching is “overlearned,” the hand motion's initiation will be easier and more reliable possibly due to stronger synaptic connections requiring less neuromodulation. In effect, what is being trained might be the overcoming of an apraxia or dyspraxia rather than the subject of the teaching. Praxic disability has been shown to be common is ASD (145, 146).

A model for this might be the symptom of freezing found in Parkinson's disease. Several authors have noted similarities between ASD and Parkinson's symptoms including freezing (147–151). Freezing in Parkinson's is when walking becomes hesitant and may come to a stop despite the intention to walk forward. It has been described by the subjects as if their feet are stuck to the floor (152). Freezing has been found not to be associated with bradykinesia and rigidity found in Parkinson's, but rather appears to have an association with decreased input from the prefrontal areas (152). In addition, there are many environmental factors which are known to affect freezing, including stress, turning direction and going into confined spaces (even virtually). Various visual or attentional cues can help alleviate freezing and it is likely that this is because the cues help to stimulate frontal lobe functioning (152). It has been found that in Parkinson's patients with freezing, there is aberrant neuronal activation in the subthalamic nucleus which is a part of the fronto-basal ganglia circuitry (153). This circuitry is responsible for the initiation or the stopping of movements as well as thought and cognition, attention impulses, emotions and complex actions (154, 155) and there is evidence that these circuits are regulated by NE as well as dopamine (156, 157). In Parkinson's disease a metronome “cue” was used to help normalize gait. It has been suggested that this is working through the basal ganglia to the prefrontal cortex and facilitates executive functioning (158) so the “prompt” is, in a sense modulating the prefrontal circuitry.

In ASD there is both a failure to initiate, as evidenced by prompt dependence, as well as a failure to inhibit or dampen many functions including motor (tics, hyperactivity), emotions, repetitive thoughts, and activities similar to that seen in obsessive compulsive disorder and impulsivity (155). Again, in ASD we can conceptualize the deficits to be as a result from poor regulation of this circuitry rather than the circuits being intrinsically dysfunctional.

## Language Acquisition

Prior to the DSM 5, a disorder of language was one of the three criteria for the diagnosis of ASD. This was eliminated due to the finding that it was neither specific nor universal (159). One can safely say however, that language disturbance is common in ASD and it is of a highly variable nature and is a very significant symptom for those affected (160). As opposed to acquired language deficits in which an already formed language ability is interrupted, in developmental disorders the study of what leads to the deficits is considerably more difficult (161). This is especially true for ASD in which there are many other cognitive and social issues which generates questions of primary or secondary status of the language deficit. There are however, very interesting leads concerning LC/NE functioning and language development from animal models.

For several decades the songbird species has been the only experimentally tractable animal model for investigating the neural substrate for the formation and function of a specialized, socially-sensitive neural circuitry for vocal learning (162). Vocal learning, the capacity to learn to produce new sounds for communication, is a rare trait across animal groups with humans and songbirds being the best characterized exemplars. Song learning and human language acquisition share several key features (163, 164). In both songbirds and humans, vocal learning (1) takes place during a critical developmental phase comprised of partially overlapping stages of sensory acquisition and sensorimotor practice; (2) relies upon social interactions and; (3) requires specialized brain circuitry dedicated to the development and production of new sounds. Once a “template” of the song, taught by a tutor, (usually the birds father) the young bird must practice the sounds using auditory feedback dependent on dopamine (165). The initial vocalizations are highly variable and unstable, akin to the babbling of human babies. It is through the social interaction with the tutor that the songs are perfected. Passive exposure to speech leads to minimal vocal learning in both humans and songbirds (166) and so the social deficits seen in ASD could be central in the deficits in language acquisition. Many of the same tactics are used by the songbirds and humans when tutoring speech, sometimes termed “motherese,” with slower speech, longer pauses between word boundaries, more repetitive speech, and higher pitch with more variable pitch. All of this may be to facilitate attention. Midbrain neurons that synthesize NE and dopamine encode social and sensory info, and modulate attention and sensory learning. It has been proposed that midbrain catecholaminergic neurons integrate multimodal sensory information derived from the social interactions with tutors and release catecholamines into brain areas important for auditory learning (165, 166). NE has been found to enhance the processing of birdsongs in auditory areas critical for song learning, thus encoding the tutor's songs (167, 168). NE enhances the coding accuracy of higher auditory cortical neurons for complex vocalizations, an effect similar to neuroestrogen modulation but without directly impacting neuroestrogen synthesis (167) and so it appears NE has an independent and specific role. NE caused a decrease in spontaneous firing (before auditory presentation) which increases signal to noise ratio, facilitating the processing of the vocalizations. These findings have been similar in birds and mammals. With little current understanding of why subjects with ASD or other developmental disorders do or do not develop language, these songbird findings may provide a valuable clue.

## Treatment Strategies

With no etiologic based treatments on the horizon for ASD, it is sensible to focus on symptomatic relief. Biologic treatments need to compliment the behavioral and educational treatments which have shown benefit. To create these biological treatments, targets must be established. Focus on the neuromodulation of the brain of individuals with ASD could be a powerful strategy. This should not be surprising as virtually all of the medications used in psychiatry function as neuromodulators (although not always characterized that way), operating on the serotonin, dopamine acetylcholine and GABA, as well as norepinephrine mediated systems. Through recognition of the neuromodulatory mechanisms for the symptoms, treatments can be devised.

In ASD noradrenergic medications commonly used include the alpha 2 agonists, clonidine (169), and guanfacine (170), the beta-adrenergic blockers including propranolol (171, 172), and the noradrenergic agonist stimulant medications (173). There is literature to support the benefit of these medications despite the dearth of rigorous study of these medications. There is no literature studying alpha 1 agonists or antagonists despite basic research which might make these agents attractive (128, 174, 175).

Despite the potential of noradrenergic agents to offer benefit, one difficult predicament is the fact that the neuromodulatory deficits noted in ASD are primarily a timing issue. As outlined above, the inability of the brain to respond to the changing demands of the environment is what must be remedied. A medication which either facilitates or inhibits the neuromodulator can have utility especially if the individual has a hyper or hypo functioning adrenergic system. However, a more complete solution would be the improved regulation of the LC. A promising modality might be external stimulation to improve neuromodulation including electrical, magnetic, ultrasound, or others. Transcranial magnetic stimulation (TMS) has thus far shown promise (176, 177). Transcranial Direct Current Stimulation (tDCS) also has shown some early promise (178–182). This treatment provides a subthreshold current to a targeted part of the brain, changing resting potentials but not triggering action potentials, thus doing part of the work toward the recruitment of the circuit desired. In theory, this would help facilitate what might have been an inadequate intrinsic neuromodulation. Providing this stimulation in tandem with education or training can lead to facilitation of the recruitment of specific circuits. A more ambitious goal would be to facilitate circuits which regulate the intrinsic neuromodulators which could have broader treatment effects.

## Conclusion

Adherence to a rigid medical model, focusing on an etiologic based target, combined with a rigid adherence to the “gold standard” of proof which is the randomized placebo controlled double blind trial, has yielded little relief for those affected by psychiatric disorders, including ASD, over the past quarter century (8, 10). This would suggest that a new outlook on the disorders and new methods of treatment study is timely.

The midbrain neuromodulators, including norepinephrine are involved with a large and diverse set of functions. Individuals with ASD also demonstrate many symptoms well-beyond the “core symptoms” which define ASD. It would seem unlikely that these symptoms have unrelated mechanisms. An outlook focused on explaining the breadth of symptoms present in ASD, including both core symptoms and those termed comorbidities is more likely to yield answers as to what truly exists in this disorder.

Inadequate neuromodulation provides a plausible explanation for many of the symptoms observed. Evidence suggests that many of the deficits observed can be conceptualized as state conditions and could be more easily treatable than the more commonly held model of deficits in hard neuronal wiring. The focus on circuitry, and the concept of neuromodulation to maximize the functioning of the circuitry, allows for the possibility of significant improvement in the lives of affected individuals with our currently available tools. To accomplish this, the research community will need to reorient thinking toward the biology of how to maximize circuit functioning which might require the outlook of personalized medicine. This may require alternative methods of research focused more on single cases studies and observational research along with alternative research models such as the RDoC program. Rather than a single minded reliance on behaviors as endpoints for treatment success or failure, new measurements of modulatory efficiency need to be explored. The study of neuromodulation takes on an even more urgent role in toddlers and very young children as inefficient neuromodulation can lead to dysfunctional circuit formation making remediation that much more difficult.

## Author Contributions

The author confirms being the sole contributor of this work and has approved it for publication.

### Conflict of Interest Statement

The author declares that the research was conducted in the absence of any commercial or financial relationships that could be construed as a potential conflict of interest.
